# Explaining transformer-based classification of radiology reports

**DOI:** 10.1093/bjrai/ubag001

**Published:** 2026-01-16

**Authors:** Megan Courtman, Galaleldin Abdelhalim, Lingfen Sun, Emmanuel Ifeachor, Stephen Mullin, Mark Thurston

**Affiliations:** Peninsula Medical School, University of Plymouth, Plymouth, Devon PL4 8AA, United Kingdom; Department of Medical Imaging, University Hospitals Plymouth NHS Trust, Plymouth, Devon PL6 8DH, United Kingdom; School of Engineering, Computing and Mathematics, University of Plymouth, Plymouth, Devon PL4 8AA, United Kingdom; School of Engineering, Computing and Mathematics, University of Plymouth, Plymouth, Devon PL4 8AA, United Kingdom; Plymouth Institute of Health and Care Research, University of Plymouth, Plymouth, Devon PL4 8AA, United Kingdom; Department of Medical Imaging, University Hospitals Plymouth NHS Trust, Plymouth, Devon PL6 8DH, United Kingdom

**Keywords:** natural language processing, radiological reporting, deep learning, transformers, explainable AI, small vessel disease

## Abstract

**Objectives:**

Deep learning models developed for the classification of radiological reports have lacked explainability. We aimed to validate and explain a pretrained classification model by applying it to the removal of confounding data from a radiological dataset.

**Methods:**

Two radiologists categorized 2038 anonymized MRI head free-text radiology reports for abnormality and for small vessel disease presence. Of these reports, 80% (*n* = 1630) were used to fine-tune pretrained transformer models to classify scans. Five-fold cross-validation was used in model development. The models were tested on the remaining 20% of the reports (*n* = 408). SHapley Additive exPlanations (SHAP) were used to explain the results.

**Results:**

The models exhibited excellent classification performance, with a mean receiver operating characteristic (ROC) area under the curve (AUC) of 0.98 for abnormality classification and 0.99 for small vessel disease classification. SHAP highlighted relevant words in both cases.

**Conclusions:**

This application validated the use of a pretrained transformer in detecting confounding data in research cohorts, and exhibited explainable results that allow the models’ decisions to be understood. By highlighting the specific report terms that drive each prediction, the explainable model output can be reviewed and critiqued by subject matter experts, supporting trust, error analysis, and iterative refinement of AI tools within clinical workflows.

**Advances in knowledge:**

This application demonstrates the feasibility of explainable report classification, and the fine-tuned model could be used in future for automatic removal of confounding data from radiology datasets, while providing transparent, case-level justifications that support audit, governance, and clinician acceptance.

## Introduction

Images and associated metadata for radiological studies form a major source of data within the electronic health record, and have the potential to support both clinical care and health research.^1^ Although structured reporting templates are being increasingly used, radiology reports are still frequently recorded as narrative free text, which can make them difficult to interpret and analyse computationally.^2^ To convert reports into a more accessible format, studies have made use of natural language processing (NLP): a technique that allows structured information to be extracted from free text.^3^ Extracted structured data has been used for a variety of purposes, such as diagnosis surveillance, case retrieval, quality assessment, patient prioritization, and research cohort selection.^1^^,^^2^^,^^4^ This field has expanded in recent years due to significant developments in deep learning techniques.^2^ Prior to this, rule-based NLP techniques were used, which were effective for radiology report classification but required extensive manual development.^5^

Various deep learning models have been trained to classify radiology reports. Long short-term memory (LSTM) networks have been applied to the classification of chest CT reports.^6^ Convolutional neural networks (CNNs) and recurrent neural networks (RNNs) have been applied to the classification of pulmonary embolism findings.^7^^,^^8^ More recently, transformer architectures have been explored, with state-of-the-art results reported for lung cancer classification amongst other tasks.^9–12^ Our study makes use of one such transformer model, originally trained on millions of radiology reports, with the aim that downstream applications might benefit from the representations learned. This model outperformed baseline transformers in tasks such as abnormal sentence classification, report coding, and summarization.^13^

Notably absent from these previous applications of deep learning to radiology reports is explainability. Explainable AI seeks to address the issue of the “black box” nature of deep learning techniques, which has inhibited their interpretability and trustworthiness.^14^ The number of studies looking at using explainable AI in healthcare has increased exponentially over the last few years, as studies have sought to make promising deep learning applications more trustworthy and viable in the healthcare setting.^15^ In applications of deep learning to radiology, explainability will be needed to audit systems, enhance trust, and adhere to regulations.^16^ It is crucial that applications of deep learning to radiological reports also adhere to these necessarily high standards.

The context for this study is a wider project investigating the use of supervised machine learning for the early diagnosis of Parkinson’s disease (PD) on the basis of routinely collected MRI imaging data. We hypothesized that NLP could be used to automatically flag potentially confounding studies during cohort construction, especially those with reports featuring small vessel disease or non-PD pathology, as these findings may mask or mimic subtle imaging signatures of early PD. A transformer-based architecture was chosen as it reflects the current state-of-the-art in radiology NLP and provides a flexible basis for extending this work beyond binary classification to richer, disease-specific labelling. This work leverages the power of both transfer learning and explainable AI to validate and explain a pretrained transformer model.

## Methods

### Subject inclusion

Ethical approval was granted on July 8, 2021, by Health Research Authority and Health and Care Research Wales. Data were obtained from a large teaching hospital with regional neuroscience services. The study design was retrospective and observational using pre-existing data.

A database of PD patients was used to identify cases for inclusion in the study. The radiology information system (RIS) was used to identify all MRI head imaging for PD patients. A custom database query was then used to search the RIS for matched controls. For each scan from a PD patient, 2 control scans were identified. All scans had been routinely acquired for any number of reasons. Scans were matched according to:

- scan type
- age at time of scan, within a window of ±6 months
- scan date, within a window of ±12 months
- biological sex

The final dataset contained a total of 2038 reports: 705 for scans from PD patients and 1333 control scans (Table 1). A non-identifiable unique identifier was assigned to each report.

**Table 1.** Demographic summary of PD and control cohorts.

Cohort	Count	Median age (interquartile range)	Male/female percentage split
Parkinson’s	705	69 (62–79)	62/38
Control	1333	70 (64–76)	61/39
Combined	2038	70 (63–76)	62/38

Abbreviation: PD = Parkinson’s disease.

### Ground truth confirmation

Manual review of reports was performed by 2 radiologists. Reference guidance on report labelling was produced to promote consistent technique between members of the labelling team. In the event of any disagreement of the correct labels, a third member of the clinical research team reviewed the case, and a discussion was held between the 3 reviewers to identify a consensus.

The task was formulated as a multi-class classification problem. Each report was given a label for abnormality. The 3 labels given were “normal” (*n* = 350), “abnormal” (*n* = 1531), or “not enough information” (*n* = 157). A demographic summary is provided in Table 2.

**Table 2.** Demographic summary of reports by abnormality classification.

Classification	Count	Median age (interquartile range)	Male/female percentage split	PD/control percentage split
Normal	350	63 (54–70)	61/39	45/55
Abnormal	1531	71 (66–77)	62/38	32/68
Not enough information	157	68 (62–72)	63/37	34/66

Abbreviation: PD = Parkinson’s disease.

Each report was also given a label for small vessel disease. The 3 labels given were “no small vessel disease” (*n* = 1009), “small vessel disease” (*n* = 869), or “not enough information” (*n* = 160). Small vessel disease was isolated for its own category as it was by far the most common pathological finding in the dataset. It is known to be a common finding in brain imaging, particularly in elderly subjects.^17^ A demographic summary is provided in Table 3.

**Table 3.** Demographic summary of reports by small vessel disease classification.

Classification	Count	Median age (interquartile range)	Male/female percentage split	PD/control percentage split
No small vessel disease	1009	67 (57–73)	61/39	32/68
Small vessel disease	869	74 (69–79)	62/38	38/62
Not enough information	160	68 (63–73)	62/38	32/68

Abbreviation: PD = Parkinson’s disease.

### Split

80% of the reports (*n* = 1630) were used to train and develop models. Five-fold cross-validation was used, with the data divided into 80% training data and 20% validation data in each fold. The 5 final developed models were tested on the remaining holdout set containing 20% of samples (*n* = 408).

### Preprocessing

Text was minimally preprocessed by replacing newline characters with spaces. Punctuation and common function words were retained, consistent with standard practice for transformer-based language models that operate on sub-word tokenizations of near-raw text. Dates and signatures were removed using an automated process.

### Model architecture

Python-based deep neural networks were built with Keras^18^ using the TensorFlow backend.^19^ A pretrained transformer and tokenizer were loaded from the Hugging Face library.^20^ RadBERT-RoBERTa-4m was trained on 4 million deidentified medical reports from the US Department of Veterans Affairs health care systems, and has previously exhibited high performance in medical language processing, with reported accuracies of over 97.5% for abnormality classification.^13^ It is built on the previous bidirectional encoder representations from transformers (BERT)^21^ and robustly optimized BERT pretraining approach (RoBERTa)^22^ algorithms. The final layers were fine-tuned for this task, and dropout of 0.5 was included before the final layer.^23^

### Model training

The models were trained for a maximum of 30 epochs using stochastic gradient descent with the Adam optimization algorithm (learning rate 0.00001).^24^ Early stopping with a patience of 20 epochs was used.^25^ A balanced sparse categorical cross-entropy loss function was utilized. The labels were encoded as integers using the LabelEncoder from scikit-learn, which does not introduce any ordinal relationship between labels.^26^

### Model assessment

The receiver operating characteristic (ROC) area under the curve (AUC) was calculated by generating a one-vs-the-rest ROC curve per class, and then taking the mean of the 3 one-vs-the-rest AUCs. As there is an imbalance in data classes (see Tables 2 and 3), the metric of balanced accuracy was reported. This is the arithmetic mean of sensitivity and specificity, and is useful when dealing with imbalanced data, as using the accuracy metric alone might exaggerate the discriminatory ability of the model.

### Explainability

SHapley Additive exPlanations (SHAP) were used to explain the models’ predictions. SHAP uses the game theory concept of Shapley values to calculate the contribution of a factor to a machine learning model output.^27^ In this case, SHAP was used to calculate and visualize the contribution of individual words to the models’ predictions. To make the explainability more accessible, SHAP values were aggregated by word (rather than the sub-word tokens used as input to the model).

## Results

### Abnormal scans

In the training set, the final models achieved a mean test ROC AUC of 0.98 and a mean test balanced accuracy of 0.88 (Table 4). When tested on the holdout set, the 5 final models achieved a mean ROC AUC of 0.98 and a mean balanced accuracy of 0.89 (Table 4).

**Table 4.** Performance metrics for the models developed to classify radiology reports.

		Mean receiver operating characteristic (ROC) area under the curve (AUC) [95% CI]	Mean balanced accuracy [95% CI]
Abnormality	Training	0.98 [0.97, 0.98]	0.88 [0.85, 0.91]
	Holdout	0.98 [0.98, 0.98]	0.89 [0.87, 0.91]
Small vessel disease	Training	0.98 [0.97, 0.99]	0.90 [0.87, 0.94]
	Holdout	0.99 [0.98, 0.99]	0.93 [0.91, 0.94]

### Small vessel disease

In the training set, the final models achieved a mean test ROC AUC of 0.98 and a mean test balanced accuracy of 0.90 (Table 4). When tested on the holdout set, the 5 final models achieved a mean ROC AUC of 0.99 and a mean balanced accuracy of 0.93 (Table 4).

### SHAP plots

#### Abnormal scans

**Figure 1.**
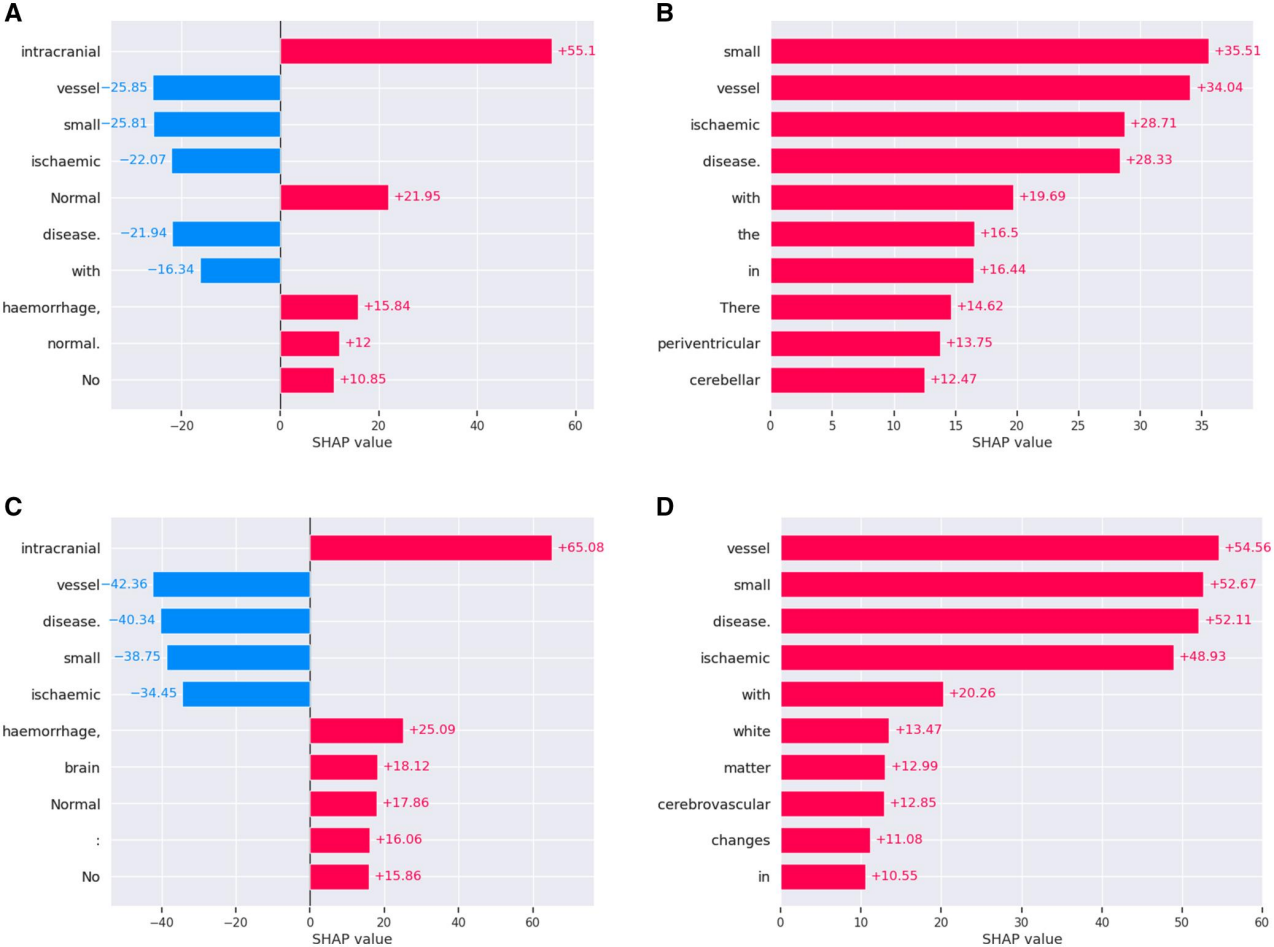
(A-D) SHAP value plots; red bars indicate positive contributions, blue bars negative contributions. (A) Normal label. (B) Abnormal label. (C) No small vessel disease label. (D) Small vessel disease label. Abbreviation: SHAP = SHapley Additive exPlanations.

**Figure 2.**
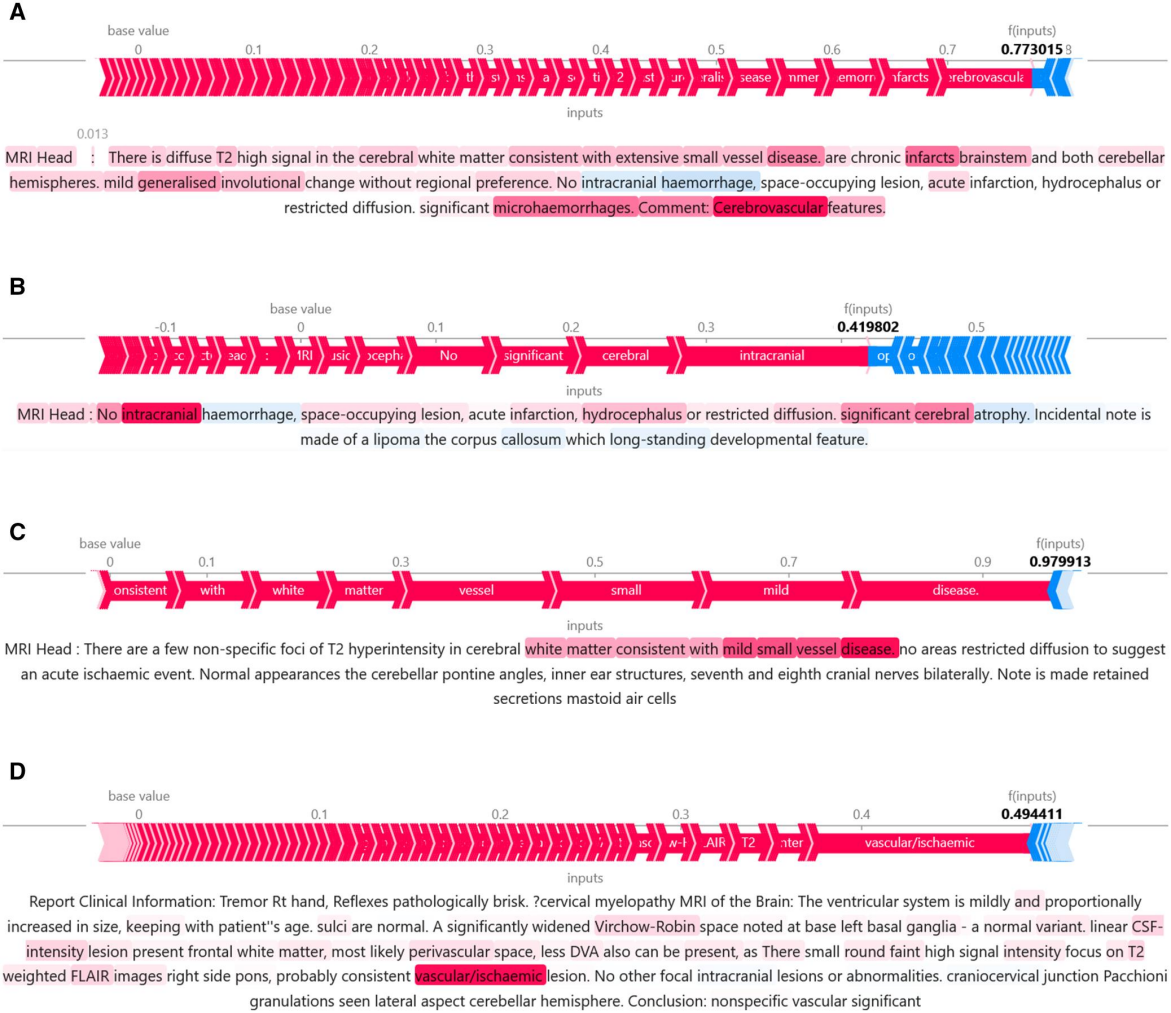
Example reports with SHAP explanations. Words in red contributed positively to the prediction; words in blue contributed negatively. (A) Abnormal report correctly classified. (B) Abnormal report misclassified as normal. (C) Small vessel disease report correctly classified. (D) No small vessel disease report misclassified as small vessel disease. Abbreviation: SHAP = SHapley Additive exPlanations.

SHAP was used to explain the models’ predictions in the holdout set. The most informative words for the “normal” class are shown in Figure 1A. The most informative words for the “abnormal” class are shown in Figure 1B. Of the 408 reports, 336 (82%) were correctly classified by all 5 models (an example is shown in Figure 2A), and 13 (3%) were misclassified by all 5 models (an example is shown in Figure 2B).

#### Small vessel disease

SHAP was used to explain the models’ predictions in the holdout set. The most informative words for the “no small vessel disease” class are shown in Figure 1C. The most informative words for the “small vessel disease” class are shown in Figure 1D. Of the 408 reports, 364 (89%) were correctly classified by all 5 models (an example is shown in Figure 2C), and 7 (2%) were misclassified by all 5 models (an example is shown in Figure 2D).

## Discussion

The deep learning models demonstrated excellent performance in classifying both abnormality and small vessel disease in radiology reports. The SHAP explainability method highlighted that relevant words were being used by the models to make these predictions. In both cases, the most informative words are often related to small vessel disease, with “small,” “vessel,” and “disease” featuring prominently, as well as “ischaemic.” This was to be expected, with small vessel disease being such a common finding in the dataset. The example shown in Figure 2A demonstrates that other less common findings can inform the models’ prediction of abnormality, which was unsurprising given that the original model was trained to classify abnormal sentences. The explainability method also provides evidence that the models are sensitive to negation, with the word “No” appearing as one of the most informative words for both “normal” and “no small vessel disease” reports. Indeed, the incorrect classification of the report shown in Figure 2B indicates that the models can occasionally be over-sensitive to negation, as the model has seemingly applied the “No” to the second sentence as well as the first. The incorrect classification of the report shown in Figure 2D is also understandable, with too much weight given to the phrase “vascular/ischaemic.” In future work, it would be informative to complement word-level SHAP explanations with approaches that directly probe the model’s use of sequential and relational information, such as phrase-level attribution or analysis of attention weights. These explanations of incorrectly classified examples provide the potential to improve the models’ performance. Future work could consider how the models might be tuned to be less prone to these types of errors. As this particular application is intended to exclude confounding data from research datasets, future work could also consider tuning the models to increase sensitivity to particular abnormalities dependent on the pathophysiology of the disease under investigation.

A limitation of this study is the lack of external validation of the fine-tuned models, as similar datasets are difficult to obtain. We have mitigated this limitation as far as possible in this study by reserving an unseen holdout test set. However, as these data originate from the same source as the training data, the metrics reported may not be representative of the models’ performance on data from a different distribution. For example, reporting styles are likely to vary between institutions. Our models are trained on narrative-style reports and are therefore most directly applicable to similar free-text corpora. Generalizability of the models to other institutions would need to be assessed by external validation, which might reveal that optimal multi-institutional performance requires further training of models on a more diverse range of reporting styles. Additionally, these data were drawn from a population of PD patients and matched controls, resulting in a dataset that is demographically unrepresentative of a general population, as there are more male than female patients and PD commonly presents later in life.^28^ These scans are likely to represent the spectrum of pathology in that age group, but the older population means that there is likely to be more pathology present in this dataset than in a more random selection of scans.

Nonetheless, the application does provide an explainable validation of the RadBERT-RoBERTa transformer. The demonstrated accuracy of the technique demonstrates its usefulness for automated processing of radiology data as part of an image analysis pipeline, allowing automated removal of investigations that may contain confounding data. In the context of the PD project, for example, these classifications could be used to automatically remove confounding “abnormal” or “small vessel disease” scans, to investigate whether this data cleaning improves the performance of PD classification models. In the future, this technique may alleviate the need for costly ground-truthing of scans by researchers.

## Conclusion

The developed NLP models had a balanced accuracy of 0.89 in classifying abnormality and 0.93 in classifying small disease presence in radiology reports, and the explainability method demonstrated that the models were focusing on appropriate words. This particular application could be used in the future for automatic removal of confounding data from radiology datasets, alleviating the need for costly ground-truthing of scans by researchers. The application also demonstrates the power of combining transfer learning and SHAP to produce a high-performance and explainable transformer model.
